# TAM Receptors Support Neural Stem Cell Survival, Proliferation and Neuronal Differentiation

**DOI:** 10.1371/journal.pone.0115140

**Published:** 2014-12-16

**Authors:** Rui Ji, Lingbin Meng, Xin Jiang, Naresh Kumar CVM, Jixiang Ding, Qiutang Li, Qingxian Lu

**Affiliations:** 1 Department of Ophthalmology and Visual Sciences, University of Louisville School of Medicine, Louisville, Kentucky, 40202, United States of America; 2 Department of Anatomical Sciences and Neurobiology, University of Louisville School of Medicine, Louisville, Kentucky, 40202, United States of America; 3 Department of Radiation Oncology, The First Hospital of Jilin University, Changchun, 130021, China; 4 Birth Defects Center, University of Louisville School of Dentistry, Louisville, Kentucky, 40202, United States of America; Indiana University School of Medicine, United States of America

## Abstract

Tyro3, Axl and Mertk (TAM) receptor tyrosine kinases play multiple functional roles by either providing intrinsic trophic support for cell growth or regulating the expression of target genes that are important in the homeostatic regulation of immune responses. TAM receptors have been shown to regulate adult hippocampal neurogenesis by negatively regulation of glial cell activation in central nervous system (CNS). In the present study, we further demonstrated that all three TAM receptors were expressed by cultured primary neural stem cells (NSCs) and played a direct growth trophic role in NSCs proliferation, neuronal differentiation and survival. The cultured primary NSCs lacking TAM receptors exhibited slower growth, reduced proliferation and increased apoptosis as shown by decreased BrdU incorporation and increased TUNEL labeling, than those from the WT NSCs. In addition, the neuronal differentiation and maturation of the mutant NSCs were impeded, as characterized by less neuronal differentiation (β-tubulin III^+^) and neurite outgrowth than their WT counterparts. To elucidate the underlying mechanism that the TAM receptors play on the differentiating NSCs, we examined the expression profile of neurotrophins and their receptors by real-time qPCR on the total RNAs from hippocampus and primary NSCs; and found that the TKO NSC showed a significant reduction in the expression of both nerve growth factor (NGF) and brain-derived neurotrophic factor (BDNF), but accompanied by compensational increases in the expression of the TrkA, TrkB, TrkC and p75 receptors. These results suggest that TAM receptors support NSCs survival, proliferation and differentiation by regulating expression of neurotrophins, especially the NGF.

## Introduction

Neurogenesis takes place in adult central nervous system in many vertebrates including human [1]. The multipotent NSCs are located in the in the subgranular zone (SGZ) of hippocampal dentate gyrus and the subventricular zone (SVZ) of the lateral ventricles [2]. The glial fibrillary acidic protein (GFAP)-positive radial glia-like cells in those regions are considered as primary stem cells normally remaining in the quiescent state, but have capacity for self-renewal and multipotential differentiation. Once activated, they develop into proliferating intermediate progenitor cells and the undifferentiated neuroblasts that will further maturate into dentate granule cells in hippocampus or interneurons in the olfactory bulb, accordingly [3], [4]. These newly generated neurons are capable to incorporate into the existing neural circuitry and contribute to brain functions [5]. Such adult neurogenesis event is a dynamic process and modulated by a variety of intrinsic and extrinsic factors including growth factors and cell surface receptors, signal transduction molecules, transcriptional factors and cytokine/chemokines [6]. Interruption of adult neurogenesis leads to impairment in hippocampus-dependent learning and behavior [6]–[12]. Many physiological and pathological conditions affect neurogenesis in adult brains. Infection and the invoked inflammation inhibit NSC proliferation and neuronal differentiation [8], [13].

Inflammation has been recognized as a major negative impact on adult neurogenesis [8], [13]. We have recently shown TAM receptors are all expressed by astrocytes and microglia, and they play an important role in regulating brain inflammation. It was found that hyperreactive microglia in the *Tyro3^−/−^Axl^−/−^Mertk^−/−^* triple knockout (TAM TKO) mice produced increased level of proinflammatory cytokines that are detrimental to the neural stem cell proliferation and differentiation [14]. However, detailed comparison of β-tubulin III^+^ neurons showed a significantly decreased neuronal differentiation from the TKO NSCs than those from the WT NSCs that had been treated with LPS-treated microglia-conditioned medium. In addition, in vivo studies on the LPS-induced inhibition of NSC proliferation and differentiation demonstrated that the adult TKO brains manifested even severer reduction in neurogenesis than the WT brains that had undergone the LPS-induced inflammation [14]. These data imply that TAM receptors might play an intrinsic functional role in NSC proliferation and neuronal differentiation.

Tyro3, Axl and Mertk belong to the structurally and functionally closely-related TAM family of receptor tyrosine kinases, expressed on the cell surface and playing divergent functional roles ranging from cell differentiation to cell death [15]. Both Gas6 and Protein S serve as ligands for this family of receptors [16]–[18]. Although originally cloned from many fast growing or transformed cells, TAM receptors are now considered as intrinsic growth trophic factors. They sustain cell growth and survival, support PC12 cell neuronal differentiation upon neuronal growth factor stimulation [19]. Genome-wide analysis of the genes differentially expressed between neuronal progenitor and the differentiated neuronal cells revealed that all three members of the TAM family are expressed in the embryonic cortical neuronal progenitor cells [20]. Mice lacking both *Axl* and *Mertk* caused early differentiation and migration of SVZ NSCs [20], and knockout of their common ligand, *Gas6*, reduced the NSC numbers in the SVZ [21]. These evidences indicate that TAM receptors might play important roles in maintenance of the cortical neuronal progenitor cell identity, in regulation of the NSCs survival, proliferation, and differentiation.

In the present study, we demonstrated that the primary NSCs express all three members of the TAM receptors to provide trophic support for themselves to ensure the survival, proliferation and differentiation into immature neurons in vitro. Under normal culture condition, TKO NSCs showed a significant reduction in NGF expression, accompanied by compensational increases in the expression of TrkA, TrkB and TrkC, suggesting that the TAM receptors function in coordination with neurotrophins in NSCs. Intrinsic trophic support by the TAM receptor signaling pathway on the NSCs may represent a novel signaling pathway in adult neural stem cells maintenance and differentiation.

## Materials and Methods

### Animal

The *Tyro3^−/−^Axl^−/−^Mertk^−/−^* triple knockout (TKO) mice have been described previously [22], [23]. All animals were housed in a pathogen-free facility and the Institutional Animal Care and Use Committee (IACUC) at University of Louisville specifically approved this study, No. 10131.

### Reagents and materials

The NSC culture supplements B-27 (100x) and N-2 (50x) were purchased from Invitrogen (San Diego, CA). The antibodies used for Western blotting and immunocytochemistry were goat anti-mouse Axl, -Tyro3 (Santa Cruz) or -Mertk (R&D), mouse monoclonal anti-mouse β-tubulin III (anti-TUJ-1, StemCell Technologies, Canada), rabbit anti TrkA (Millipore: 06-574), Rat anti NGF (Abcam: ab52918), anti-mouse GFAP (EMD Millipore, MA), rat monoclonal anti-BrdU (Novus Biologicals, CO), mouse monoclonal anti-mouse β-actin (Sigma-Aldrich), HRP-conjugated donkey or sheep polyclonal anti-goat/mouse IgG (Amersham, NJ) antibodies and used following the manufacturer’s instructions.

### RNA isolation, cDNA synthesis, and real-time quantitative PCR

Total RNAs from cultured NSC cells, and the hippocampus of 8-week-old WT and TKO mice were extracted using RNeasy kit (Qiagen) or Trizol reagent (Invitrogen, CA), respectively, following the manufacturer’s instructions. The integrity of the RNA samples was determined by 1% formaldehyde denaturing RNA agarose gels (18S/28S = 2∶1) and by O.D. reading (A260/A280>1.9). Two micrograms of total RNA from each sample was treated with DNase I to remove traces of genomic DNA, followed by reverse transcription using qScript cDNA SuperMix kits (Quanta Biosciences, Gaithersbur, MD) to make the first strand cDNA for real-time qPCR analysis.

A sample of 50 ng of cDNA was used to quantify gene expression by qPCR using a SYBR green-based PCR reaction mixture on an MX3005p system (Agilent Technologies, Inc., Santa Clara, CA), with a program of a 10-minute initial hot-start activation of *Taq* polymerase at 95°C, followed by 40 cycles of amplification (95°C for 10 seconds, 56°C for 5 seconds, and 72°C for 10 seconds). After amplification, a melting curve was generated by holding the reaction mixture at 65°C for 15 seconds, then heating to 95°C at a ramp rate of 0.1°C/s. To obtain the melting temperature for each sample, the fluorescence signal was plotted against temperature. The comparative CT (threshold cycle) method normalized to β-actin was used to analyze relative changes in gene expression.

The oligonucleotides used for qPCR are listed in table 1.

**Table 1:** The oligonucleotides used for qPCR.

Oligo	Forward (5′-3′)	Reverse (5′-3′)
*BDNF*	AGCTGAGCGTGTGTGACAGT	ACCCATGGGATTACACTTGG
*NT3*	CTACTACGGCAACAGAGACGCT	GGTGAGGTTCTATTGGCTACCAC
*NGF*	TCCACCCACCCAGTCTTCCA	CCTTCCTGCTGAGCACACA
*TrkA*	ACGGTAACAGCACATCAAGAG	GGAGGGCAGAAAGGAAGAG
*TrkB*	AAGGACTTTCATCGGGAAGCTG	TCGCCCTCCACACAGACAC
*TrkC*	CAACTCTCAAACACGGAGGTC	CCAGCATGACATCGTACACC
*P75*	CGTATTCCGACGAGGCCA ACC	CCACAAGGCCCACAACCACAGC
*β-actin*	GGCTGTATTCCCCTCCATCG	CCAGTTGGTAACAATGCCATGT

### Isolation and primary culture of neural stem cells

For isolation of brain stem cells, newborn mice at postnatal day 0 (P0) or P1 were terminated by decapitation and at least 3 forebrains from each group of mice were carefully dissected, pooled together in a 5 ml conical tube containing 500 µl of Dulbecco’s modified Eagle’s medium (DMEM)/F12 medium (Invitrogen), and sliced into small pieces. After 5 min of settling, the supernatant was gently removed and the remaining tissue was incubated with 500 µl of 0.25% trypsin/0.03% EDTA at 37°C for 3 min followed by addition of 500 µl of soybean inhibitor (1 mg/ml) to inactivate the trypsin. After gentle trituration with a 1 ml pipette, the cell suspension was centrifuged at 250×g for 5 min at room temperature, the supernatant was carefully removed afterwards, and the cells were washed with 1 ml of complete NSC medium [DMEM/F12, 1xB-27, 1xN-2, 20 ng/ml of bFGF, 20 ng/ml of EGF, 1x penicillin/streptomycin (Invitrogen)] and filtered through a 100-micron cell strainer to obtain the single cell suspension. After centrifugation at 250xg for 5 min at room temperature and removal of the supernatant, the cell pellets were resuspended in 1 ml of complete NSC medium. Viable cells were counted by the Evans Blue staining. Depending on the type of experiment, 0.5 to 1×10^5^ cells in 500 µl of complete NSC medium were plated into each well of a 12-well plate or 0.5–1×10^5^ cells in 2 ml of complete NSC medium were plated into each well of a 6-well plate; in both cases, the proliferating neural stem cells formed NSC spheres in 4 days.

### Long-term survival, proliferation, and differentiation of NSCs

To test long-term survival, the single cell suspension of WT or TKO NSCs (2×10^5^ cells in 2 ml per well) was plated on 6-well plates. When the cells had grown to approximately 200 µm in diameter (4–5 days), the NSC spheres were dissociated using 0.25% trypsin/10 mM EDTA and a small amount of sample was taken for cell number counting. The remaining single cell suspension was then plated as described above. This repeated digestion/plating keeps the proliferating cells in a healthy condition and can be performed for up to 10 cycles before the NSCs started to differentiate. The total cell number at each time point was calculated based on the cells number from each counting and the fraction of the cells used for next cycle of subculture. The day for primary cell culture was designated as P0 and the cell number was set as 1. The growth curve was plotted by total cell number against the days in culture.

### *In* *vitro* differentiation, proliferation, and death of NSCs

The single cell suspension of NSCs was cultured for 5 days in the complete NSC medium. Once the neuroblast spheres had grown to approximately 150–200 µm in diameter, they were switched to NSC differentiation medium and continue to incubate at 37°C for 5 days to induce cell differentiation.

For neuronal differentiation from the single cell suspension, the NSC spheres were first triturated into single cells after 1 min of digestion with 0.05% trypsin at 37°C. The dissociated single NSC cell suspension (10^5^ cells/ml in 400 µl of differentiation medium) was plated in 8-well cell culture chambers, which had been coated by 50 µg/ml of poly-L-ornithine and 5–7 µg/ml laminin. Then the cells were cultured for 60 hrs. in the differentiating medium (complete NSC culture medium supplemented with 10%FBS and 10% Differentiation Supplements [StemCell Technologies]). The differentiated cells were fixed for 20 min at room temperature in 4% paraformaldehyde (PFA) prior to immunostaining using antibodies specific for β-tubulin III, glial fibrillary acidic protein (GFAP), and staining of nuclei with Hoechst 33342.

For the NSC proliferation assay, NSC spheres were labeled with 10 µM BrdU for 12 hrs, followed by 3 times washes with phosphate-buffered saline (PBS). The cells were collected by centrifugation, and fixed for 2 hrs at 4°C in 4% PFA. After a brief centrifugation, the sphere pellets were collected and embedded in 100 µl of melted 2% agar containing 5% formalin. After solidification, the neuroblast spheres in agar were embedded in OCT (Optimal cutting temperature compound), frozen in liquid nitrogen, and cryrosectioned at a thickness of 18 µm. Incorporated BrdU was immunostained with anti-BrdU antibody and nuclei counterstained with Hoechst 33342.

To measure NSC survival, the TUNEL assay was performed using the Roche TUNEL labeling kit (Indianapolis), following the manufacturer’s instructions.

### Western blotting

For expression of TAM receptors in NSCs, NSCs were isolated from hippocampi of the WT and TKO mice at age of postnatal day-1 and the same number of cultured NSC in 6-well plate was directly lysed in 1X NuPAGE LDS sample buffer (Invitrogen, CA) and the cell lysates were separated by SDS-PAGE electrophoresis, transferred onto a nitrocellulose membrane and immunoblotted for Tyro3, Axl, Mertk, and β-actin. The NSC cell lysate from TKO mice was used as negative control and the β-actin was used to indicate the sample loading. For expression of NGF and TrkA in hippocampus, hippocampal lysates were derived from 2 months old mice and immunoblotted for NGF, TrkA and β-actin.

### Immunocytochemistry

NSC spheres or the differentiated single neuronal cells on chamber slides were processed for histology as above. The sections or cells were blocked in 1xPBS plus 0.1% Triton X-100 and 3% normal serum for 1 hr at RT, and followed by incubation at 4°C for overnight in primary antibodies (1∶200 dilution in blocking buffer). After 5 washes (5 min for each wash) in 1xPBS plus 0.1% Triton X-100, the sections or cells were incubated in the fluorochrome-conjugated secondary antibodies (1∶500 dilution in 1xPBS) for 1 hr at RT. After 5 washes, the slides were mounted in DAPI-containing medium (Vectashield, Vector Laboratories). Photographs were taken on a Carl Zeiss AxioImager M2 cell imaging microscope equipped with Apotome and AxioCam systems (Carl Zeiss).

### Statistical analysis

Data were analyzed using analysis of variance (ANOVA), the unpaired *t* test was used for two group comparisons and ANOVA Tukey’s multiple comparison tests were used for analysis of differences in three or more groups. All experiments were performed at least three times in triplicate. Data are expressed as the mean±SD.

## Results

### TAM receptors are expressed in hippocampal neural stem cells

**Figure 1:**
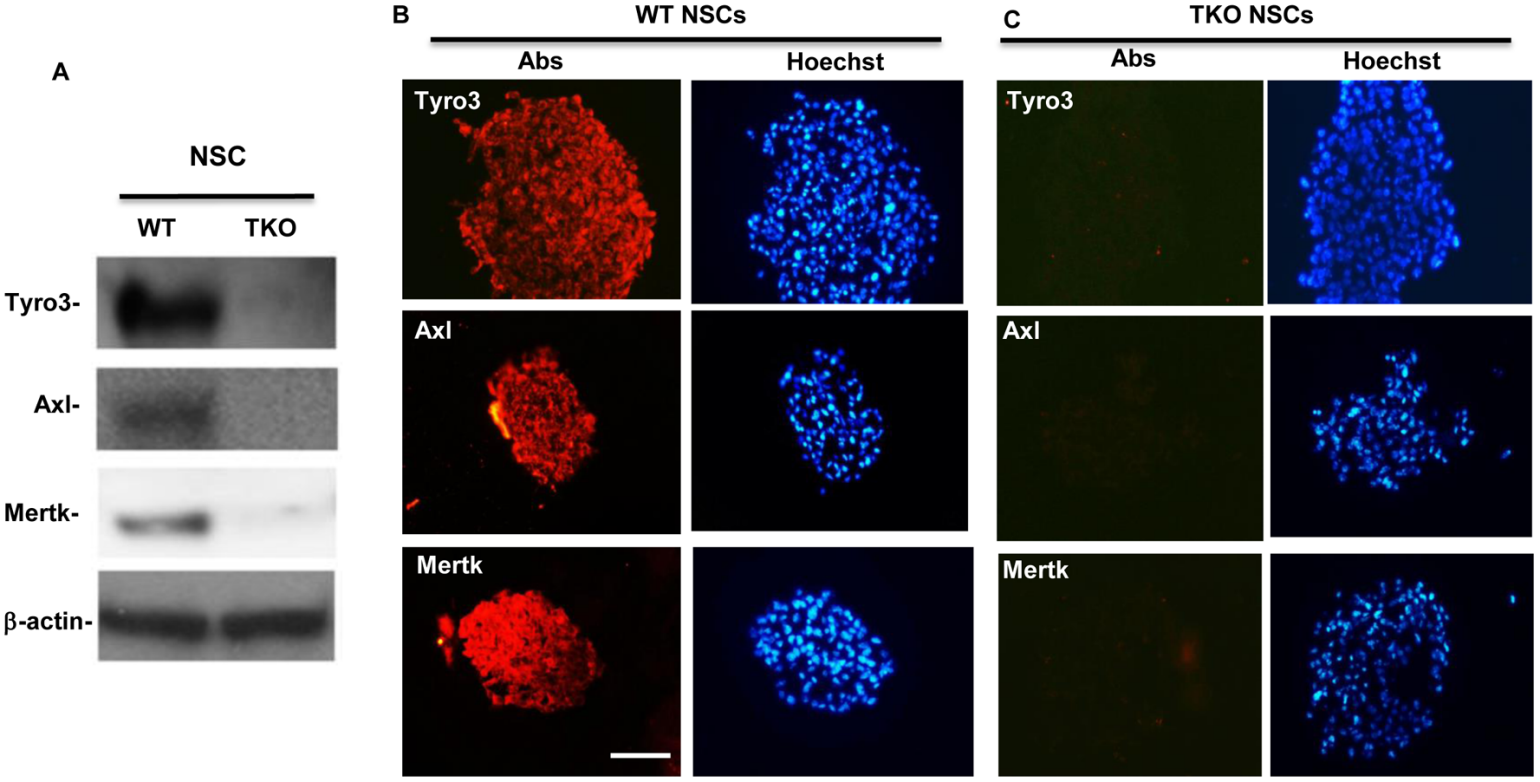
Tyro3, Axl, and Mertk receptors are expressed in primary hippocampal NSCs. (A) Examination of TAM receptors expression in hippocampal NSCs by western blotting. (B and C) NSC spheres were obtained and processed for immunohistochemistry following the procedures described in the Methods. The cryosections were immunostained with anti-Tyro3, -Axl or -Mertk antibodies, accordingly (B, red) and nuclei were counter-stained with Hoechst 33342 (blue). The NSC spheres from the TKO mice were used as the negative control (C). Scale bar, 100 µM representing all the scale in (B and C).

The TAM family of receptor tyrosine kinases, Tyro3, Axl and Mertk, are mainly expressed in immune, reproductive and nerve systems [22], [24]–[30]. In brain, all three receptors are expressed in hippocampus, especially in the subgranular layer of the dentate gyrus, as demonstrated by *in* *situ* hybridization [29], [30]. However, whether or not they are expressed in hippocampal neural stem cells is not clear. To determine whether the TAM receptors are expressed by neural stem cells and play intrinsic functional roles in the hippocampal NSCs, we performed Western blotting and immunostaining on the cultured primary WT NSCs. While all three proteins were negligibly seen from the TKO cells, they were clearly expressed in the WT NSCs with the strongest signal given by Tyro3 (Fig. 1A). Expression of all three receptors in NSCs was further confirmed by immunocytochemistry, in which the specific antibodies against Tyro3, Axl and Mertk, positively labeled the WT NSCs (Fig. 1B) but not the TKO NSCs (Fig. 1C).

### TAM receptors support primary NSCs growth and survival

**Figure 2:**
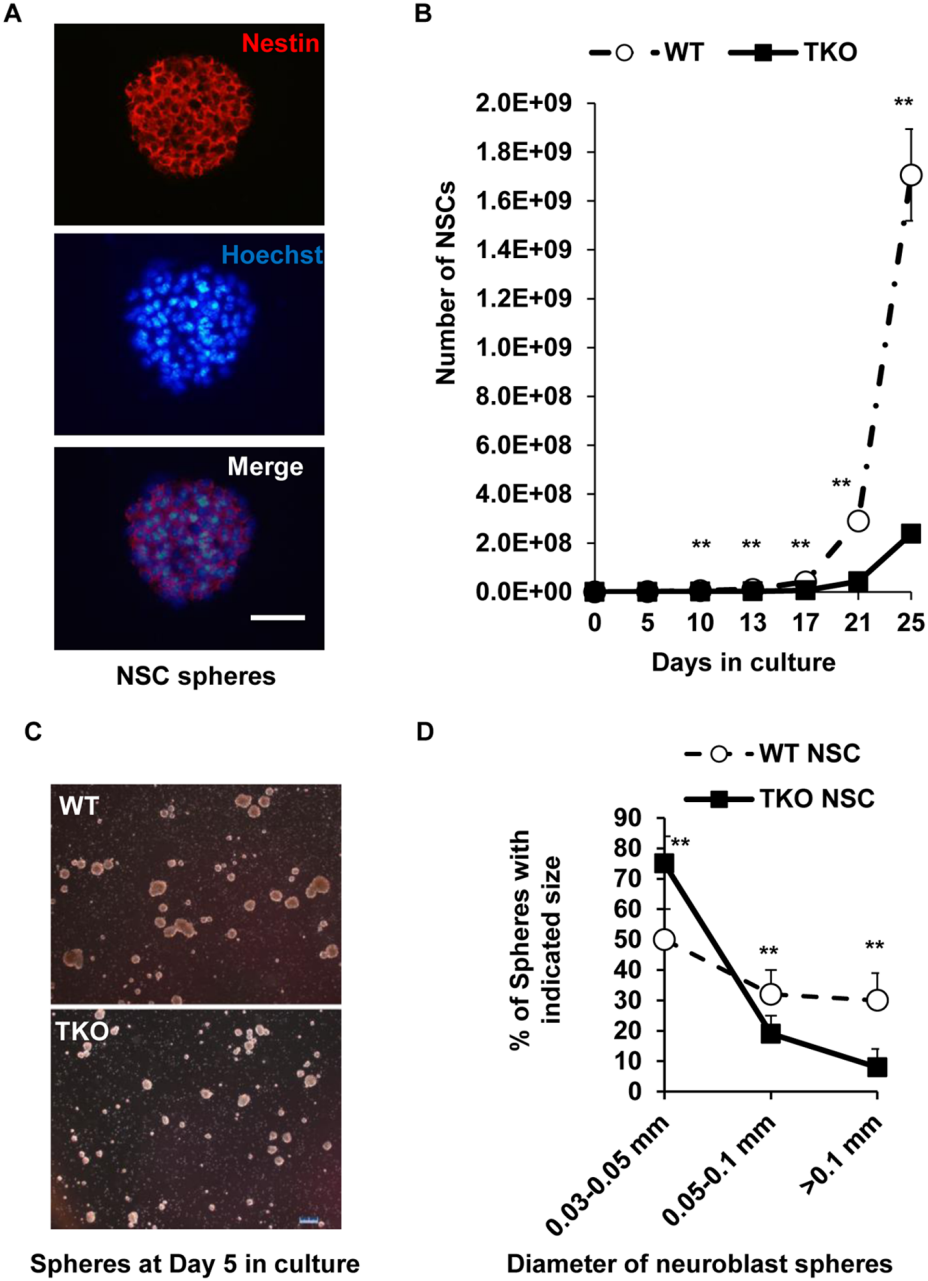
TAM receptors support primary NSCs growth and survival. (A) The WT NSC spheres were subjected to cryostat sectioning as described in the Method. The sections were immunostained using anti-nestin antibody, and nuclei were stained blue using Hoechst 33342. Scale bar, 100 µm. (B) To maintain healthy growth of the NSCs, neuronal spheres were subcultured as described in the Method. The total cell number at each time point was calculated based on the cell number from each count and the fraction of the cells used for the next cycle of subculture. The growth curves are plotted as total cell numbers against days in culture. Data are expressed as the means ± SD at each time point. **P<0.01, n = 3. (C) NSC spheres were developed from single cell suspension of both WT and TKO NSCs in 5 days and photographed, scale bar, 200 µm. (D) The newly developed spheres in each of the WT and TKO samples were designated into three groups based on the sphere sizes of large (>0.1 mm), middle (0.05–0.1 mm) and small (0.03–0.05 mm) in diameter. The number of spheres in each group was counted and the percentage of each group among the same phenotype spheres was plotted. The data are expressed as the mean ± SD of three experiments. **P<0.01, n = 3.

Expression of all three TAM receptors by the cultured primary NSCs prompted us to explore the in vitro effects of the TAM receptors on NSC growth and survival. We isolated and cultured NSCs from hippocampi of both WT and TKO mice at postnatal day one. The single hippocampal cell suspension was plated in 6-well plate and the growing NSCs gradually formed neuroblast spheres that reached approximately 200 m in diameter in five days. Most of cells in such neuroblast spheres were immunostained positive for nestin, a common neuronal progenitor marker (Fig. 2A). To sustain the proliferation and growth of NSCs, such neuroblasts were further dissociated and the resulting single cell suspension was re-plated in a new 6-well plate. The growth curves obtained using these repeated subcultures showed that both WT and TKO NSCs displayed a continuous increase in total cell number, but the TKO NSCs showed significantly slower growth than the WT cells (Fig. 2B). The significant difference between the TKO and WT groups were noticed from day-10 and thereafter. (TKO 18.5±6.3 vs WT 52.8±18.6 on day-10; TKO 33.8±13.7 vs WT 109.1±24.2 on day-13, and TKO 165.5±17.3 vs WT 417.4±67.6 on day-17, accordingly, if the starting cell number in both groups were arbitrarily designated as 1 on the day-0). Consistent with the lower growth curve of the TKO cells, the neuroblast spheres formed from TKO cells showed a lower percentage of large spheres (>0.1 mm in diameter) and fewer middle size of spheres (0.05–0.1 mm in diameter), but a higher percentage of small spheres (0.03–0.05 mm in diameter) than those formed from WT cells under the same culture conditions (Fig. 2C and 2D). These data indicate that the growth of the NSCs is hindered in the absence of the trophic support from the Tyro3, Axl and Mertk receptors.

### TAM receptors support primary NSCs proliferation

**Figure 3:**
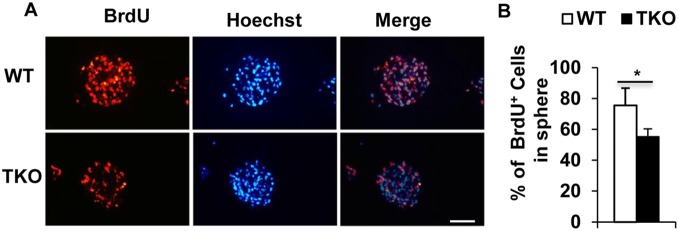
TAM receptors support primary NSCs proliferation. (A) A representative picture showing decreased BrdU incorporation into TKO NSC spheres. The proliferating stem cells were labeled with BrdU and identified with anti-BrdU antibody (red) and counter-stained with Hoechst 33342 for visualization of nuclei (blue). Scale bar, 200 m. (B) The percentage of BrdU-positive NSCs in total Hoechst stained cells are expressed as means ± SD. *P<0.05, n = 3.

To further evaluate the effects of TAM receptors on the NSC proliferation, we labeled the developing NSC spheres at day 4 using 10 M BrdU for 2 hrs. The BrdU-labeled NCS spheres were collected and processed for paraffin-embedding and sectioned at 20-µm. The BrdU incorporated into the proliferating cells were analyzed using fluorescent anti-BrdU antibody. The TKO NSCs showed lower percentage of BrdU-labeled cells than the WT cells (Fig. 3A and 3B, 55% vs 75%, *p<0.05). This result suggests that the TAM receptors support NSC proliferation *in* *vitro*.

### TAM receptors prevent primary NSCs death from apoptosis

**Figure 4:**
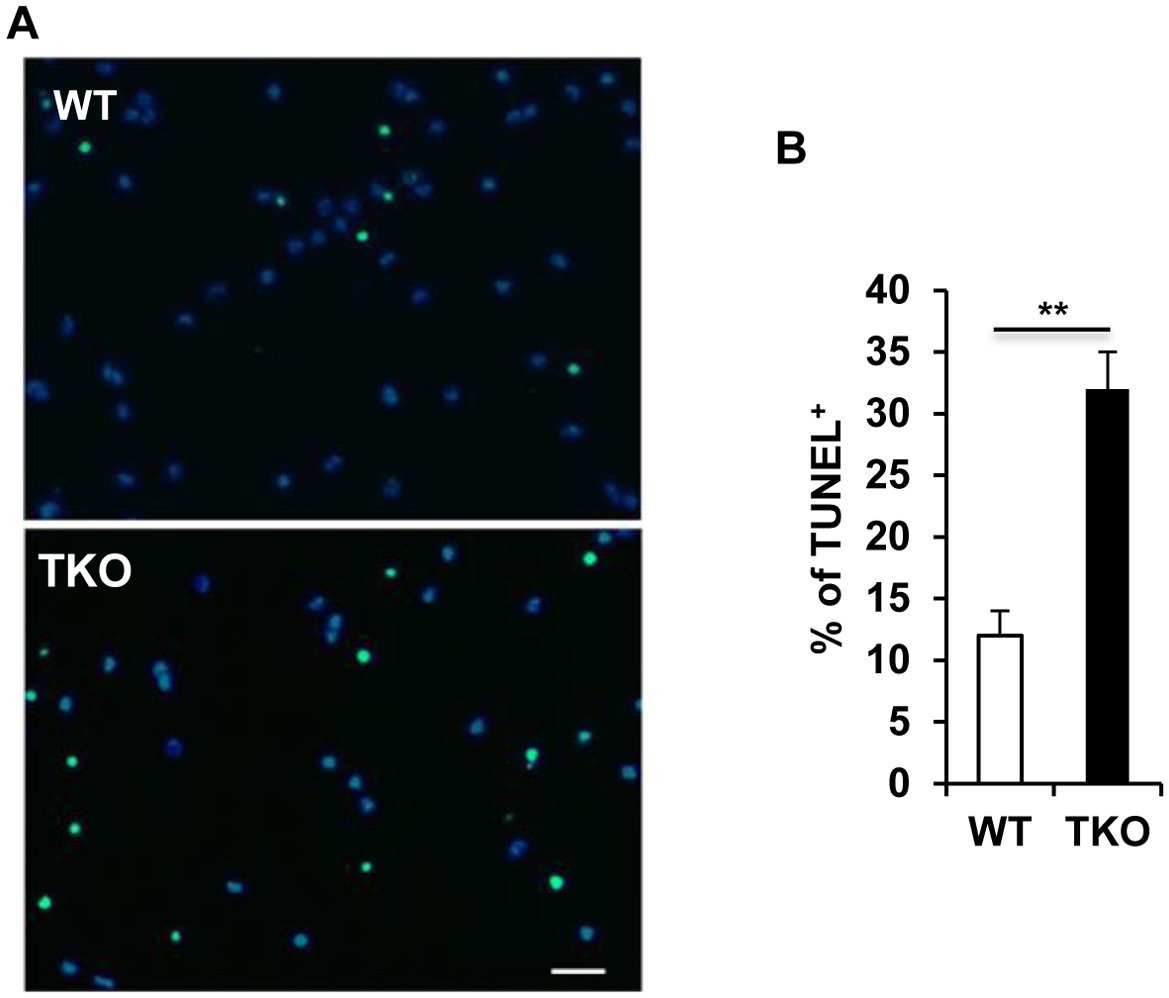
TAM receptors prevent primary NSCs death from apoptosis. The apoptotic cells in the differentiating spheres were labeled by TUNEL assay following manufacture’s instruction (Roche). Representative pictures in (A) show TUNEL staining of the differentiated WT and TKO NSCs. Scale bar, 50 µm. (B) Percentage of apoptotic cells in total differentiated NSCs. Data was presented as mean ± SD, **P<0.01, n = 3.

Our previous study showed that the ligand of TAM receptors, Gas6, protected the PC12 cells (derived from a pheochromocytoma of the rat adrenal medulla) from the serum- and NGF-deprivation induced apoptosis [19]. This prompted us to investigate whether the TAM receptors could also prevent the cultured NSCs from excessive apoptotic cell death. We therefore quantified apoptotic cells by a TUNEL assay on the primary cultured cells, and the NSCs lacking TAM receptors clearly showed increased TUNEL labeling (Fig. 4A and B), indicating a trophic support that TAM receptors provide for NSC survival *in* *vitro*.

### TAM receptors regulate primary NSCs differentiation

**Figure 5:**
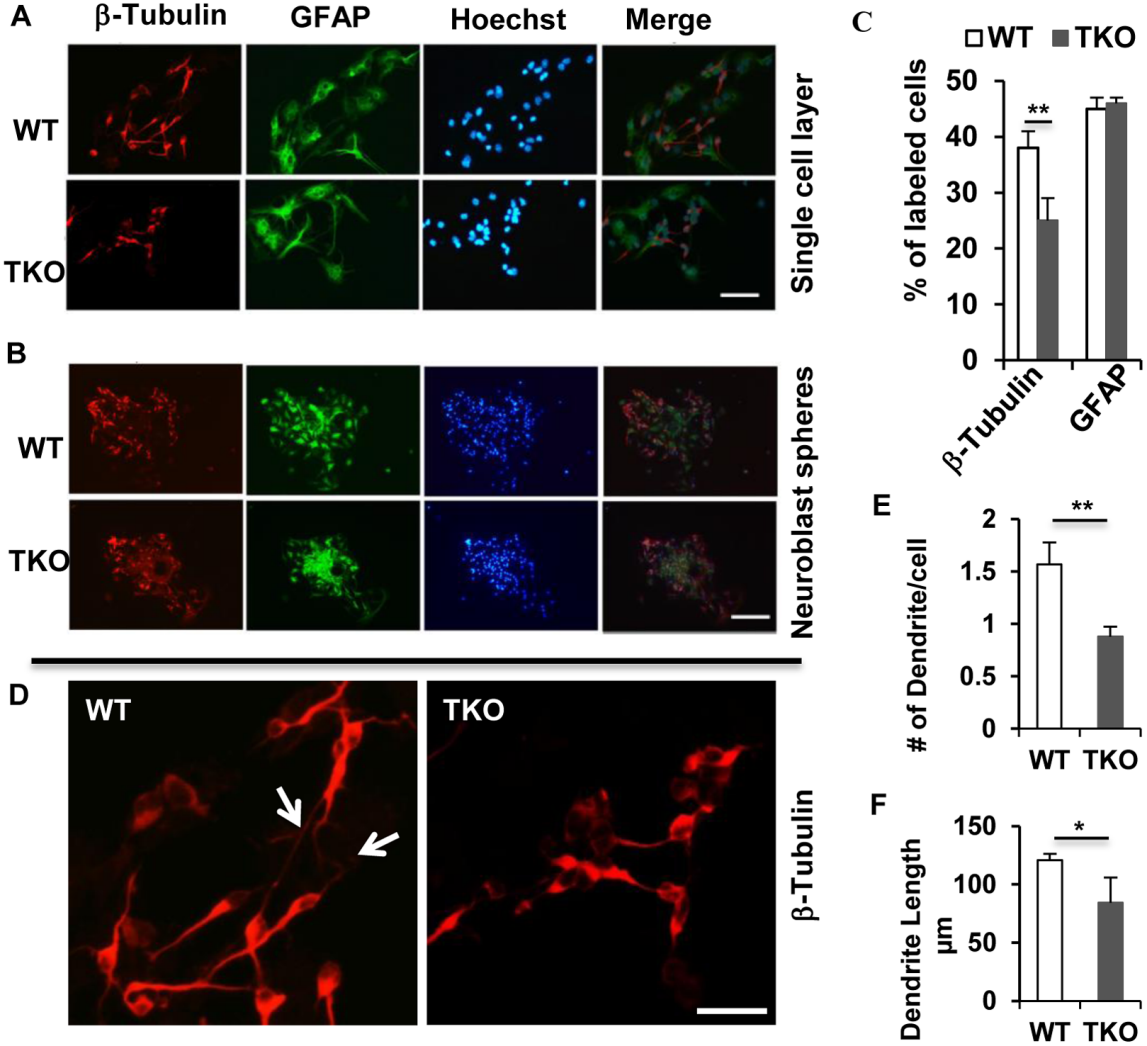
TAM receptors regulate primary NSCs differentiation. Neuronal differentiation was conducted following the procedure in the Method. The differentiated cells were identified by anti-β-Tubulin and anti-GFAP antibodies and nuclei were stained by Hoechst 33342 dye. Images were observed and the photographs were taken using a Zeiss Apotome microscope. Representative pictures show the newly-differentiated neurons (β-Tubulin) and astrocytes (GFAP) in the single cell layers (A) or neuroblast spheres (B), scale bar, 200 µm. (C) The percentage of cells expressing lineage-specific markers for neuron (β-tubulin) and astrocyte(GFAP) was plotted. Data was presented as Mean ± SD **P<0.01, n = 3. (D) The β-tubulin-III positive neurons in (A) were taken at higher magnification showing neurite outgrowth in the WT but not in the TKO cells, scale bar, 50 µm. (E) and (F) show significant difference in The number of dendrite per cell (E) and the dendrite length (F) between the WT and TKO groups were significantly different. Data was Mean ± SD, **P<0.01 and *P<0.05, n = 20.

Given that Tyro3 and Axl were associated with TrkA (the receptor for NGF) in PC12 cells and function to promote neuronal differentiation [31], we wondered whether TAM receptors would also regulate NSCs differentiation. To examine the NSCs differentiation capacity, we cultured either the trypsin-dissociated NSCs in single cell layer or the neuroblast spheres in, respectively, 8-well chambers or 24-well plates for 60 hr in NSC differentiation medium [DMEM/F12 containing 10% FBS and 10% differentiation supplements (StemCell Technologies, Canada)]. The cells in single cell layer or the spheres were then briefly washed with PBS and fixed with 4% PFA for 20 minutes prior to routine histological process. Newly-differentiated neurons and astrocytes in the single cell layers (Fig. 5A) or in the neuroblast spheres (Fig. 5B) were identified, respectively, by immunocytochemistry using antibodies against β-tubulin-III and GFAP, and the cell nuclei were labeled with Hoechst 33342 dye. The percentage of β-tubulin-III^+^ or GFAP^+^-labeled cells was calculated and the result clearly showed that the NSCs without the TAM receptors exhibited decreased differentiation into neurons, but normal differentiation into astrocytes (Fig. 5C). Interestingly, the neurite outgrowth of the newly differentiated neurons from TKO NSCs or spheres was dramatically inhibited, as compared to those differentiated from the WT NSC cells (Fig. 5D). Those results indicate that TAM receptors are required for normal differentiation of NSCs into the neuron, but likely inessential for in vitro differentiation into astrocytes.

### TAM receptors regulate expression of neurotrophins and their receptors

**Figure 6:**
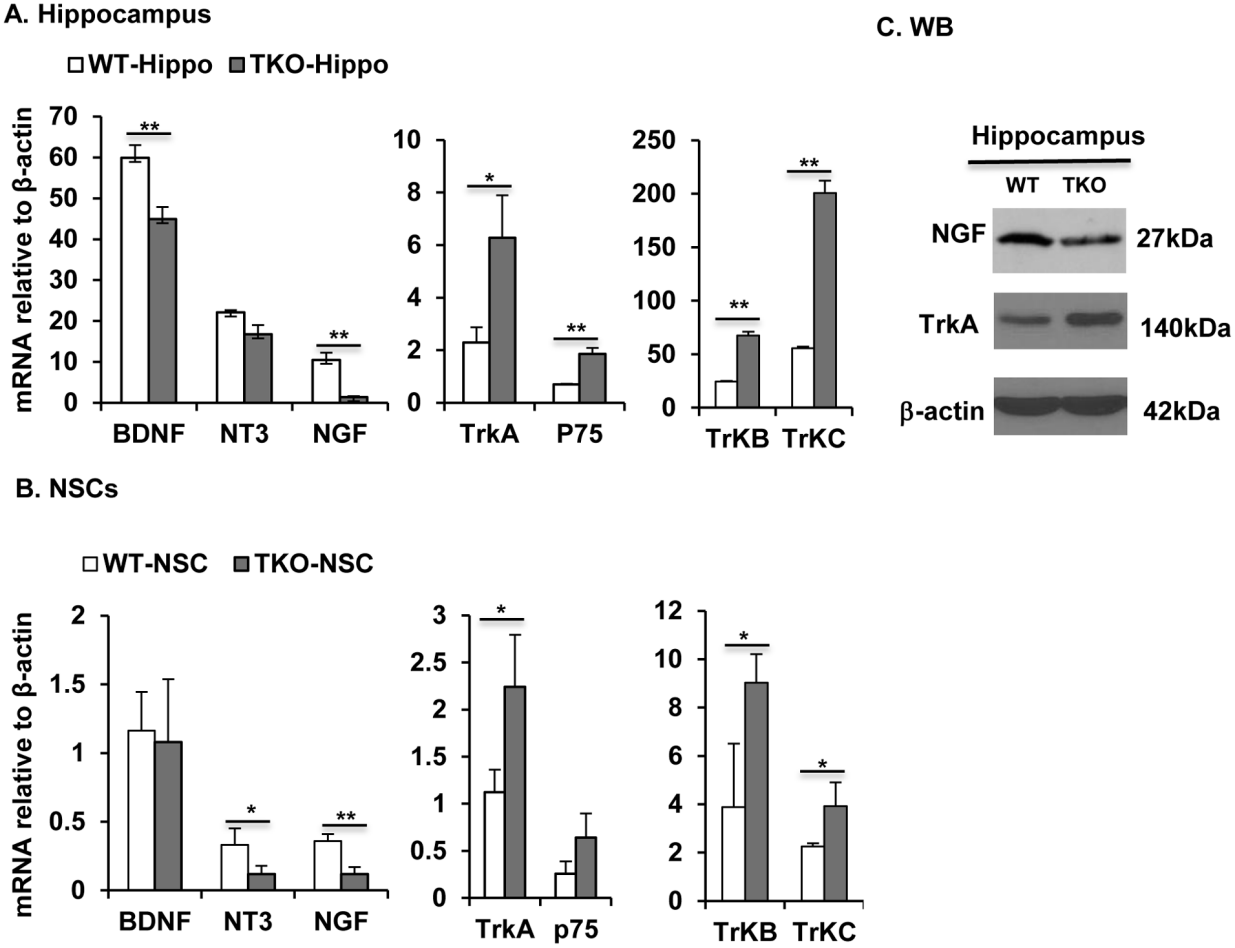
TAM receptors regulate expression of neurotrophins and their receptors. (A, B) Real-time qPCR quantification of neurotrophins, BDNF, NT-3, NGF and their receptors, TrkA, p75, TrkB and TrkC in hippocampus (A) and NSCs (B). Data are shown as means ± SD, *P<0.05 and **p<0.01, n = 3. (C) Western blotting of NGF and TrkA level in WT and TKO hippocampus. This is one representative of three mice.

Since both Tyro3 and Axl were co-localized with TrkA on the PC12 cells, and NGF up-regulated both receptors on the differentiating PC12 cells [32], we then asked whether the TAM receptors regulated NSC survival, neuronal differentiation, and neurite outgrowth through expression of neurotrophins and their receptors in the NSCs. We performed real-time-qPCR quantification of the BDNF, NT-3, NGF and their p75 and Trk receptors. The expression of both BDNF and NGF was significantly inhibited in the TKO hippocampus, with the NGF being the most affected and NT3 showing no significant change (Fig. 6A). The dramatic decreased NGF expression but not the BDNF was also found in the primary cultured adult hippocampal NSCs (Fig. 6B). Decreased expression of NGF in the mutant hippocampus was shown by Western blotting (Fig. 6C). On the other hand, their Trk family of receptors showed a significant compensational increase in both TKO hippocampus and cultured NSCs, as assayed by qPCR (Fig. 6A and 6B) and Western blotting (Fig. 6C). The TKO hippocampus also showed an increased expression of p75 (Fig. 6A). These expression changes suggest that the TAM receptors regulate NSC survival, neuronal differentiation, most likely via regulation of the neurotrophin expression, especially the NGF expression.

## Discussion

We have recently shown that mice lacking the TAM receptors displayed impaired adult hippocampal neurogenesis [14]. All three of the TAM receptors are expressed by microglia and astrocytes; and knockout of all three *Tyro3, Axl* and *Mertk* genes, caused both cell types to exhibit enhanced innate immune responses to TLR activation and to produce higher levels of proinflammatory cytokines detrimental to NSC self-renewal and neuronal differentiation [14]. In addition, several lines of evidence showed that all three receptors were expressed in hippocampus, especially in the subgranular layer of the DG, as demonstrated by in situ hybridization [29], [30]. However, whether or not they are expressed in hippocampal neural stem cells is not clear. In the present study, we performed Western blot and immunostaining analysis of Tyro3, Axl and Mertk in the primary cultured NSCs, and found that those cells expressed all three receptors. Based on these receptors’ growth trophic roles in many cell types [19], [22], [27], we hypothesized that the TAM receptors might also play intrinsic tropic functions in the NSCs. This was true that the primary cultured TKO NSCs versus their WT counterparts exhibited slower growth rate, decreased proliferation, and survival capacity, as demonstrated by poor BrdU incorporation and increased TUNEL labeling, respectively. These observations strongly suggest that TAM receptors provide trophic support for NSCs proliferation and survival. On the other hand, studies on the *Axl* and *Mertk* double knockout mice showed that embryonic SVZ NSCs in the double mutant embryos exhibited early differentiation and migration [20]. The TKO NSCs in our suspension culture system might undergo premature differentiation resulting in lower growth and proliferation rate and smaller sphere size.

Recent studies indicate that TAM receptors function in NSCs [20], [32], but whether they participate in neuronal differentiation from primary NSCs is unknown. In the present study, we showed that TAM receptors were required for NSCs differentiation into neurons, but not for the glial cell differentiation although the TAM receptors surely play important regulatory role in the astrocyte, especially under immune challenge conditions [14]. Interestingly, newly-differentiated neurons from TKO NSCs showed significant reduction in the developing neurite outgrowth, implying that TAM receptors play role in neuronal differentiation and maturation. This result is consistent with our previous observation in which the TAM ligand, Gas6, can replace the NGF to support PC12 growth and differentiation[32].

It is well documented that NGF induces neurite outgrowth and promotes survival of embryonic sensory and sympathetic neurons in culture and prevented naturally occurring cell death in developing sympathetic ganglia and cholinergic neurons of the basal forebrain and caudatoputamen [33], [34]. NGF belongs to neurotrophin family consisting of four members, namely, NGF, BDNF, neurotrophin-3 (NT-3), and neurotrophin-4/5 (NT-4/5) [35]. Each of the mammalian neurotrophins can activate one or more members of the tropomyosin-related kinase (Trk) family of receptor tyrosine kinases (TrkA, TrkB, and TrkC) or the low-affinity p75 receptor. NGF interacts with p75 and TrkA, while the BDNF and NT-4 bind TrkB, and NT-3 acts on TrkC [35]–[37]. Mice lacking Trk have severe sensory and sympathetic neuropathies and extensive neuronal cell loss during development of both the peripheral and the central nervous system [33]. Neurotrohins in collaboration with retinoic acid regulate neurogenesis in the adult-derived NSC cultures [38]. The major downstream signaling pathways activated by Trk receptors are Ras, PLC-c1, PI3-kinase (PI3K) and the mitogen-activated protein kinase (MAPK) [31], [39], [40]. Both NGF and BDNF, via activation of Trk receptors, induce activation of the PI3K/Akt and MAPK pathways, and thus protect NSC from neurotoxic induced apoptosis [41].

Our previous study showed that both Tyro3 and Axl were expressed on the PC12 cell surface and colocalized with TrkA receptor, and their ligand Gas6 could activate both PI3K, MAPK and induces neuronal differentiation and neurite outgrowth in PC12 in the absence of the NGF [32]. In the present study, we further demonstrated that the TAM receptors played intrinsic functional role in the differentiating NSCs to promote the neuronal differentiation and neurite outgrowth. This function of TAM receptors may realize through upregulation of neurotrophins, particularly the NGF, and activation of their Trk receptors. From this point of views, the TAM receptor mediated signal pathway may offer a potential candidate target for treatment of some neuropathies or neurotrophin insufficiency diseases.

## References

[1] Eriksson PS, Perfilieva E, Bjork-Eriksson T, Alborn AM, Nordborg C, et al (1998) Neurogenesis in the adult human hippocampus. Nat Med 4:1313–1317. 9809557 10.1038/3305

[2] Gage FH (2000) Mammalian neural stem cells. Science 287:1433–1438. 10688783 10.1126/science.287.5457.1433

[3] Ming GL, Song H (2011) Adult neurogenesis in the mammalian brain: significant answers and significant questions. Neuron 70:687–702. 21609825 10.1016/j.neuron.2011.05.001 PMC3106107

[4] Zhao C, Teng EM, Summers RG Jr, Ming GL, Gage FH (2006) Distinct morphological stages of dentate granule neuron maturation in the adult mouse hippocampus. J Neurosci 26:3–11. 16399667 10.1523/JNEUROSCI.3648-05.2006 PMC6674324

[5] Deng W, Aimone JB, Gage FH (2010) New neurons and new memories: how does adult hippocampal neurogenesis affect learning and memory? Nat Rev Neurosci 11:339–350. 20354534 10.1038/nrn2822 PMC2886712

[6] Zhao C, Deng W, Gage FH (2008) Mechanisms and functional implications of adult neurogenesis. Cell 132:645–660. 18295581 10.1016/j.cell.2008.01.033

[7] Monje ML, Mizumatsu S, Fike JR, Palmer TD (2002) Irradiation induces neural precursor-cell dysfunction. Nat Med 8:955–962. 12161748 10.1038/nm749

[8] Monje ML, Toda H, Palmer TD (2003) Inflammatory blockade restores adult hippocampal neurogenesis. Science 302:1760–1765. 14615545 10.1126/science.1088417

[9] Madsen TM, Kristjansen PE, Bolwig TG, Wortwein G (2003) Arrested neuronal proliferation and impaired hippocampal function following fractionated brain irradiation in the adult rat. Neuroscience 119:635–642. 12809684 10.1016/s0306-4522(03)00199-4

[10] Shors TJ, Miesegaes G, Beylin A, Zhao M, Rydel T, et al (2001) Neurogenesis in the adult is involved in the formation of trace memories. Nature 410:372–376. 11268214 10.1038/35066584

[11] Dupret D, Revest JM, Koehl M, Ichas F, De Giorgi F, et al (2008) Spatial relational memory requires hippocampal adult neurogenesis. PLoS One 3:e1959. 18509506 10.1371/journal.pone.0001959 PMC2396793

[12] Zhang CL, Zou Y, He W, Gage FH, Evans RM (2008) A role for adult TLX-positive neural stem cells in learning and behaviour. Nature 451:1004–1007. 18235445 10.1038/nature06562

[13] Carpentier PA, Palmer TD (2009) Immune influence on adult neural stem cell regulation and function. Neuron 64:79–92. 19840551 10.1016/j.neuron.2009.08.038 PMC2789107

[14] Ji R, Tian S, Lu HJ, Lu Q, Zheng Y, et al (2013) TAM Receptors Affect Adult Brain Neurogenesis by Negative Regulation of Microglial Cell Activation. The Journal of Immunology 191:6165–6177. 24244024 10.4049/jimmunol.1302229 PMC3870476

[15] Lemke G, Rothlin CV (2008) Immunobiology of the TAM receptors. Nature Reviews Immunology 8:327–336. 10.1038/nri2303 PMC2856445 18421305

[16] Stitt TN, Conn G, Goret M, Lai C, Bruno J, et al (1995) The anticoagulation factor protein S and its relative, Gas6, are ligands for the Tyro 3/Axl family of receptor tyrosine kinases. Cell 80:661–670. 7867073 10.1016/0092-8674(95)90520-0

[17] Nagata K, Ohashi K, Nakano T, Arita H, Zong C, et al (1996) Identification of the product of growth arrest-specific gene 6 as a common ligand for Axl, Sky, and Mer receptor tyrosine kinases. Journal of Biological Chemistry 271:30022–30027. 8939948 10.1074/jbc.271.47.30022

[18] Prasad D, Rothlin CV, Burrola P, Burstyn-Cohen T, Lu Q, et al (2006) TAM receptor function in the retinal pigment epithelium. Molecular and Cellular Neuroscience 33:96–108. 16901715 10.1016/j.mcn.2006.06.011

[19] Zheng Y, Zhang L, Lu Q, Wang X, Yu F, et al (2009) NGF-induced Tyro3 and Axl function as survival factors for differentiating PC12 cells. Biochem Biophys Res Commun 378:371–375. 19027714 10.1016/j.bbrc.2008.11.049

[20] Wang J, Zhang H, Young AG, Qiu R, Argalian S, et al (2011) Transcriptome analysis of neural progenitor cells by a genetic dual reporter strategy. Stem Cells 29:1589–1600. 21805534 10.1002/stem.699 PMC3262150

[21] Gely-Pernot A, Coronas V, Harnois T, Prestoz L, Mandairon N, et al (2012) An Endogenous Vitamin K-Dependent Mechanism Regulates Cell Proliferation in the Brain Subventricular Stem Cell Niche. Stem cells 30:719–731. 22290807 10.1002/stem.1045 PMC3601423

[22] Lu Q, Gore M, Zhang Q, Camenisch T, Boast S, et al (1999) Tyro-3 family receptors are essential regulators of mammalian spermatogenesis. Nature 398:723–728. 10227296 10.1038/19554

[23] Ye F, Li Q, Ke Y, Lu Q, Han L, et al (2011) TAM receptor knockout mice are susceptible to retinal autoimmune induction. Invest Ophthalmol Vis Sci 52:4239–4246. 21467176 10.1167/iovs.10-6700 PMC3175940

[24] Scott RS, McMahon EJ, Pop SM, Reap EA, Caricchio R, et al (2001) Phagocytosis and clearance of apoptotic cells is mediated by MER. Nature 411:207–211. 11346799 10.1038/35075603

[25] Lemke G, Rothlin CV (2008) Immunobiology of the TAM receptors. Nat Rev Immunol 8:327–336. 18421305 10.1038/nri2303 PMC2856445

[26] Seitz HM, Camenisch TD, Lemke G, Earp HS, Matsushima GK (2007) Macrophages and dendritic cells use different Axl/Mertk/Tyro3 receptors in clearance of apoptotic cells. J Immunol 178:5635–5642. 17442946 10.4049/jimmunol.178.9.5635

[27] Lu Q, Lemke G (2001) Homeostatic regulation of the immune system by receptor tyrosine kinases of the Tyro 3 family. Science 293:306–311. 11452127 10.1126/science.1061663

[28] Xiong W, Chen Y, Wang H, Wu H, Lu Q, et al (2008) Gas6 and the Tyro 3 receptor tyrosine kinase subfamily regulate the phagocytic function of Sertoli cells. Reproduction 135:77–87. 18159085 10.1530/REP-07-0287

[29] Prieto AL, O’Dell S, Varnum B, Lai C (2007) Localization and signaling of the receptor protein tyrosine kinase Tyro3 in cortical and hippocampal neurons. Neuroscience 150:319–334. 17980494 10.1016/j.neuroscience.2007.09.047 PMC2231337

[30] Prieto AL, Weber JL, Lai C (2000) Expression of the receptor protein-tyrosine kinases Tyro-3, Axl, and mer in the developing rat central nervous system. J Comp Neurol 425:295–314. 10954847

[31] Zheng WH, Kar S, Quirion R (2002) FKHRL1 and its homologs are new targets of nerve growth factor Trk receptor signaling. Journal of neurochemistry 80:1049–1061. 11953455 10.1046/j.0022-3042.2002.00783.x

[32] Zheng Y, Zhang L, Lu Q, Wang X, Yu F, et al (2009) NGF-induced Tyro3 and Axl function as survival factors for differentiating PC12 cells. Biochemical and biophysical research communications 378:371–375. 19027714 10.1016/j.bbrc.2008.11.049

[33] Smeyne RJ, Klein R, Schnapp A, Long LK, Bryant S, et al (1994) Severe sensory and sympathetic neuropathies in mice carrying a disrupted Trk/NGF receptor gene. Nature 368:246–249. 8145823 10.1038/368246a0

[34] Bradshaw RA, Blundell TL, Lapatto R, McDonald NQ, Murray-Rust J (1993) Nerve growth factor revisited. Trends Biochem Sci 18:48–52. 8488558 10.1016/0968-0004(93)90052-o

[35] Bibel M, Barde Y-A (2000) Neurotrophins: key regulators of cell fate and cell shape in the vertebrate nervous system. Genes & development 14:2919–2937. 11114882 10.1101/gad.841400

[36] Huang EJ, Reichardt LF (2003) Trk receptors: roles in neuronal signal transduction*. Annual review of biochemistry 72:609–642. 10.1146/annurev.biochem.72.121801.161629 12676795

[37] Chao MV (2003) Neurotrophins and their receptors: a convergence point for many signalling pathways. Nature Reviews Neuroscience 4:299–309. 12671646 10.1038/nrn1078

[38] Takahashi J, Palmer TD, Gage FH (1999) Retinoic acid and neurotrophins collaborate to regulate neurogenesis in adult-derived neural stem cell cultures. J Neurobiol 38:65–81. 10027563

[39] Middleton G, Wyatt S, Ninkina N, Davies AM (2001) Reciprocal developmental changes in the roles of Bcl-w and Bcl-x (L) in regulating sensory neuron survival. Development 128:447–457. 11152643 10.1242/dev.128.3.447

[40] Riccio A, Ahn S, Davenport CM, Blendy JA, Ginty DD (1999) Mediation by a CREB family transcription factor of NGF-dependent survival of sympathetic neurons. Science 286:2358–2361. 10600750 10.1126/science.286.5448.2358

[41] Nguyen N, Lee SB, Lee YS, Lee KH, Ahn JY (2009) Neuroprotection by NGF and BDNF against neurotoxin-exerted apoptotic death in neural stem cells are mediated through Trk receptors, activating PI3-kinase and MAPK pathways. Neurochem Res 34:942–951. 18846424 10.1007/s11064-008-9848-9

